# Acetabular paralabral cysts demonstrating perineural propagation

**DOI:** 10.1259/bjr.20211306

**Published:** 2022-07-08

**Authors:** John Hynes, Avneesh Chabra, Mina Guirguis, Eoin Kavanagh

**Affiliations:** 1 Department of Radiology, National Orthopaedic Hospital of Ireland, Cappagh, Ireland; 2 Radiology, UT Southwestern Medical Center, Dallas, Tx, United States; 3 Orthopedic Surgery, UT Southwestern Medical Center, Dallas, Tx, United States

## Abstract

**Objective:**

Acetabular paralabral cysts are common and are almost always associated with labral tears. Uncommonly, they extend into the periacetabular soft tissues or may propagate along peripheral nerves causing pain and hip dysfunction. The aim was to evaluate the clinical and MRI presentations of such cases including perineural propagation.

**Methods:**

Retrospective cross-sectional study with a search of electronic health records for cases of acetabular paralabral cysts demonstrating perineural propagation was performed. Clinical and MR imaging features were tabulated after re-review by experienced musculoskeletal radiologists, and available outcomes were recorded. Descriptive statistics were performed.

**Results:**

14 cases were recorded. The mean age was 56.9 years (range = 30–79 years) and female:male ratio was 1:2.6. The commonest presenting complaint was hip pain (10/14, 71.4%). Other complaints included groin pain, perineal pain and hip dysfunction. No symptoms were attributed to the acetabular paralabral cyst in 3/14 patients (21.4%). None had foot drop. The cysts were multilocular in all cases and were homogenously T2 hyperintense in 13/14 (92.9%). Labral tears were identified in 11/14 cases (78.6%). The sciatic nerve was most commonly involved in 5/14 cases (35.7%) with the obturator, medial femoral cutaneous nerve, femoral nerve, superior and inferior gluteal nerves also affected.

No intervention was undertaken in 9/14 cases (64.3%). 5/14 (35.7%) underwent image guided aspiration and corticosteroid injection. 4/5 such patients reported reduced pain following the procedure.

**Conclusion:**

Paralabral cysts demonstrating perineural propagation are uncommon and exhibit varied presentations. Most patients who underwent image-guided or surgical interventions reported an improvement in symptoms.

**Advances in knowledge.:**

This is the first description of a series of patients with acetabular paralabral cysts demonstrating perineural propagation in the literature. A comprehensive description of their clinical and imaging characteristics and interventions/outcomes where relevant is provided.

## Introduction

Acetabular paralabral cysts are common at the hip joint. They are almost always associated with hip labral tears and are thought to arise from increased intra-articular pressure generated during movement forcing synovial fluid through the damaged labrum and/or synovial lining into the periarticular soft tissues.^1^ They can be associated with mechanical symptoms including pain, restricted movement, hip dysfunction, and a snapping/clicking sensation at the hip.^2^ There is a relative paucity of description of these entities in the literature to date. Paralabral cysts can compress critical nerves in the vicinity or extend in retrograde fashion along articular branches of femoral, obturator, sciatic, and gluteal nerves, causing hip, groin, perineal or deep gluteal pain and dysfunction. These were the cases recorded by the authors. Femoral vascular compression due to a paralabral cyst has also been described.^3^ While hip labral variations and tear patterns have been extensively studied, paralabral cysts demonstrating perineural propagation only appear in the English literature as case reports.^2–5^ A thorough evaluation of these cysts and their appearances and presentations was the aim of this study. We aimed to evaluate and present a series of such patients and discuss their clinical presentations and MR imaging characteristics. As a secondary aim, we evaluated their treatments and outcomes.

## Methods

This was a retrospective cross-sectional study performed in a HIPAA compliant manner at two tertiary care institutions under local institutional IRB approval.

### Patients

Patient search criteria included ages 18–100 years, all genders, paralabral cysts of hip, and labral tears. Local institutional picture archiving communication systems were searched utilizing multiple search terms; ‘labral tear’, ‘cyst’, ‘hip’, perineural cyst’, ‘MR imaging’ and ‘MRI’. Relevant cases were recorded and images reviewed. Cases where paralabral cysts were considered to demonstrate perineural propagation—extending adjacent to nerves and in the same direction as the nerve—were recorded.

### Data recorded

Demographic data including age and gender was recorded. Clinical and MR imaging features were tabulated, and relevant outcomes were recorded in Microsoft Excel (Windows 11, Redwood, Seattle). Clinical data recorded included any symptoms described by the patient and whether the patient had leg weakness or foot drop. The MR imaging scans were performed on both 1.5 T (Tesla) and 3 T scanners with a combination of *T*_1_W (*T*_1_ weighted), *T*_2_W and PDW (proton density-weighted) images. MR imaging data was re-recorded by experienced musculoskeletal faculty in both institutions and included the location of the paralabral cyst, maximum size in the longest dimension (in centimetres), signal alterations on *T*_1_W and *T*_2_W/PDW MR imaging, whether the cyst was uni- or multilocular, major neurovascular structure affected, condition of labrum (degenerated or torn), and regional denervation changes. The MR imaging findings were recorded in consensus. Related interventions and outcomes, if available, were recorded. Descriptive statistics were performed, and medians and ranges were recorded.

## Results

### Demographics and clinical presentations

10 years of imaging records including over 5000 individual studies were reviewed. Only 14 cases were recorded. The patients were aged between 30 and 79, with a mean age of 56.9 years. The female:male ratio was 1:2.6. The commonest presenting complaint was hip pain in 10 of the 14 patients (71.4%). Other presenting complaints included groin pain (3/14, 21.4%), perineal pain (2/14, 14.3%), and hip dysfunction—subjective clicking and ‘locking’ in the hip (1/14, 7.1%). No symptoms were attributed to the paralabral cyst in 3/14 patients (21.4%). None had foot drop or objective leg weakness.

### MR imaging characteristics

**Figure 1.**
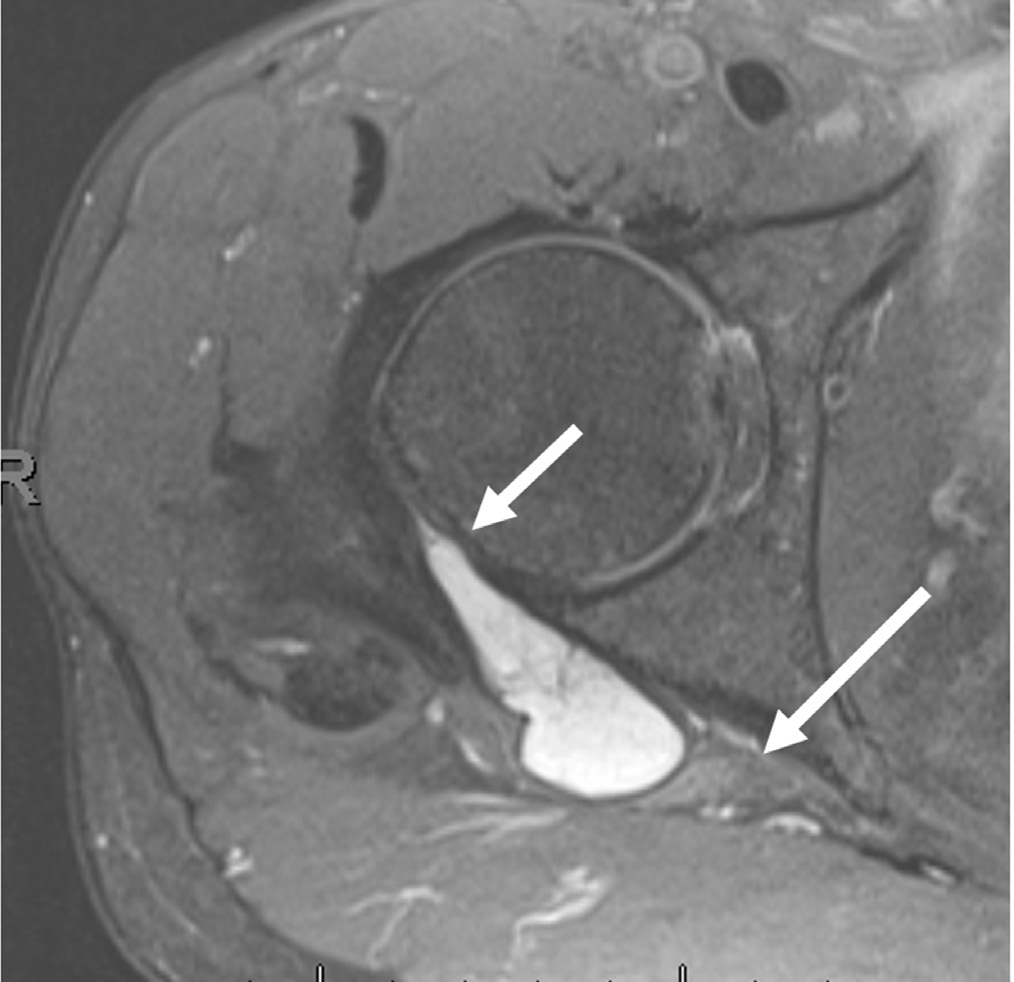
40-year-old male. Axial fsPDW demonstrates a paralabral cyst arising from the labrocapsular junction of the right hip (small arrow) with medial extension and compression of the right sciatic nerve (long arrow). fsPDW, fat saturated proton density-weighted.

**Figure 2.**
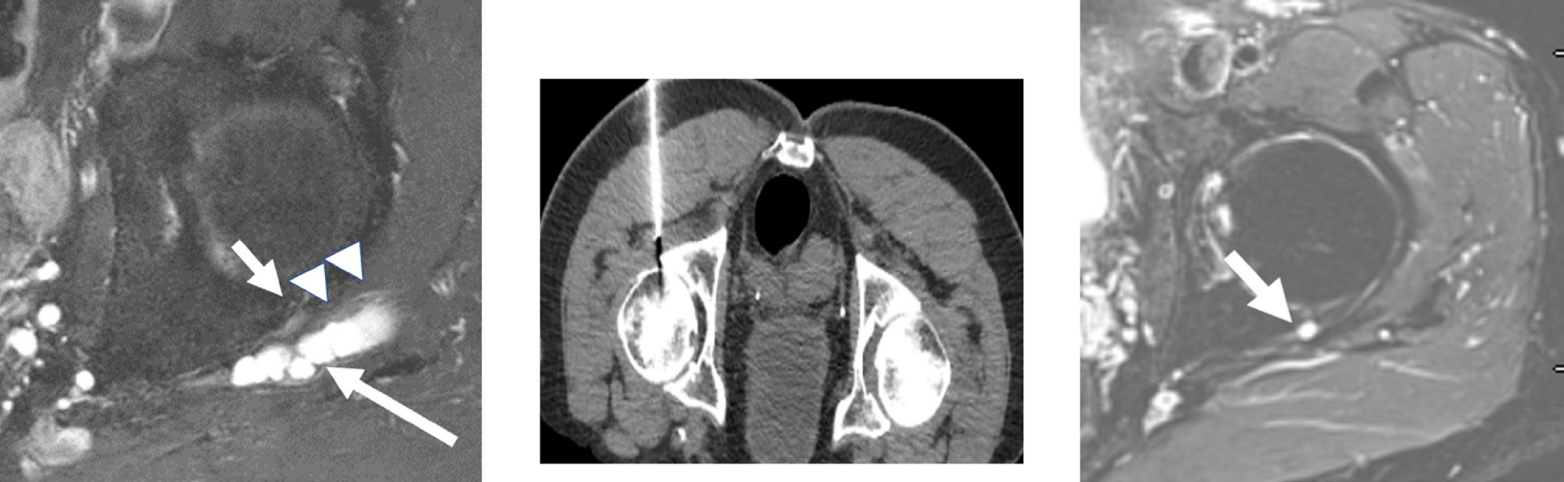
46-year-old female. Figure 2A: Axial fsPDW image demonstrating a paralabral cyst (long arrow) arising from a posterior labral tear (small arrows) with posterolateral extension adjacent to the sciatic nerve (arrowheads).Figure 2B: CT image in prone position during cyst fenestration and attempted aspiration with 16G needle. Figure 2C. Follow-up axial fsPDW MR of left hip 2 years later shows resolution of the cyst with residual labral tear still present (small arrow). fsPDW, fat saturated proton density-weighted.

**Figure 3.**
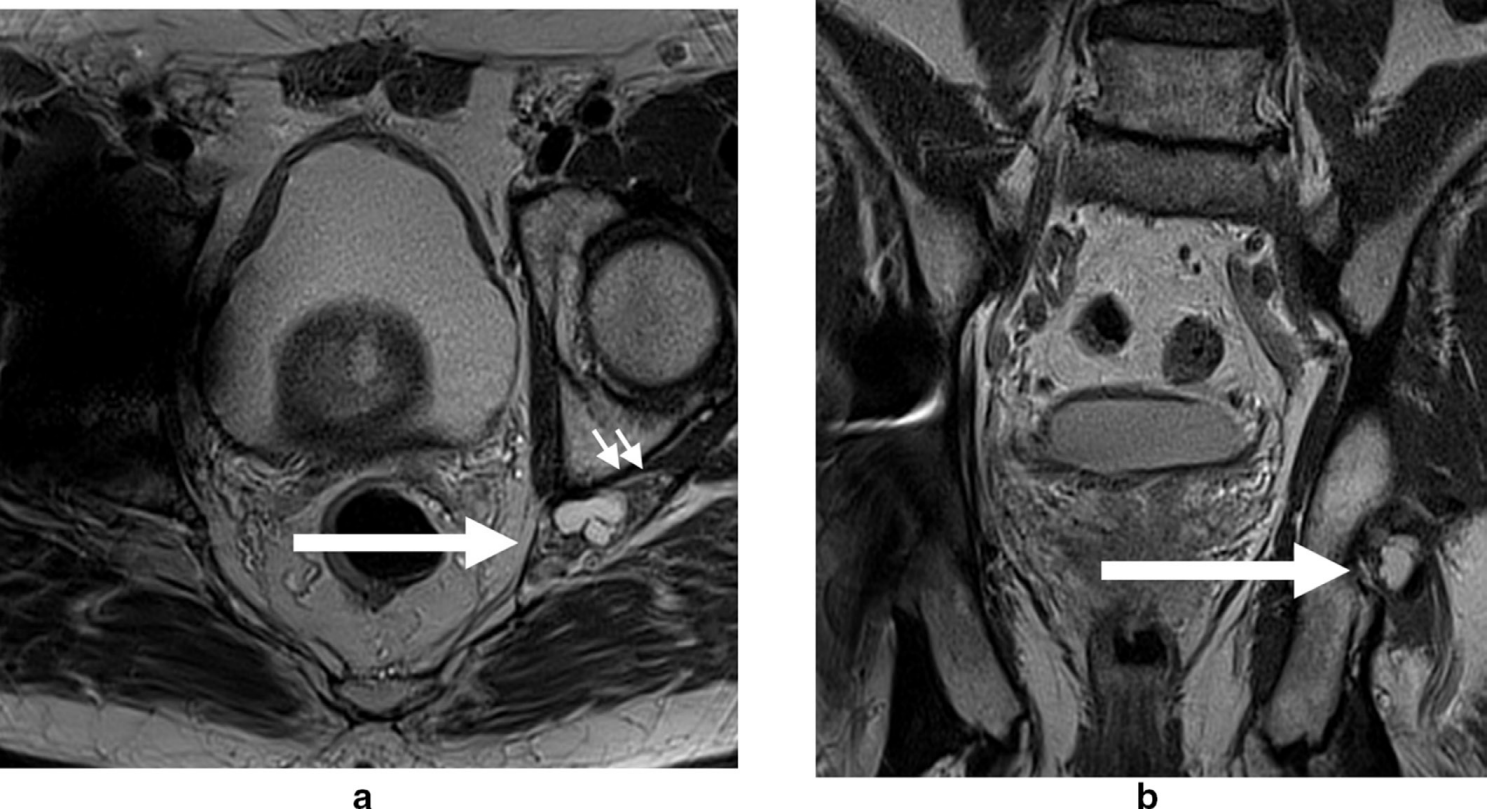
56-year-old male. Figure 3A (axial) and Figure 3B (coronal) *T*_2_W images demonstrating a paralabral cyst (long arrows) arising posteriorly from the left hip joint extending towards the sciatic nerve (short arrows). *T*_2_W, *T*_2_ weighted.

**Figure 4.**
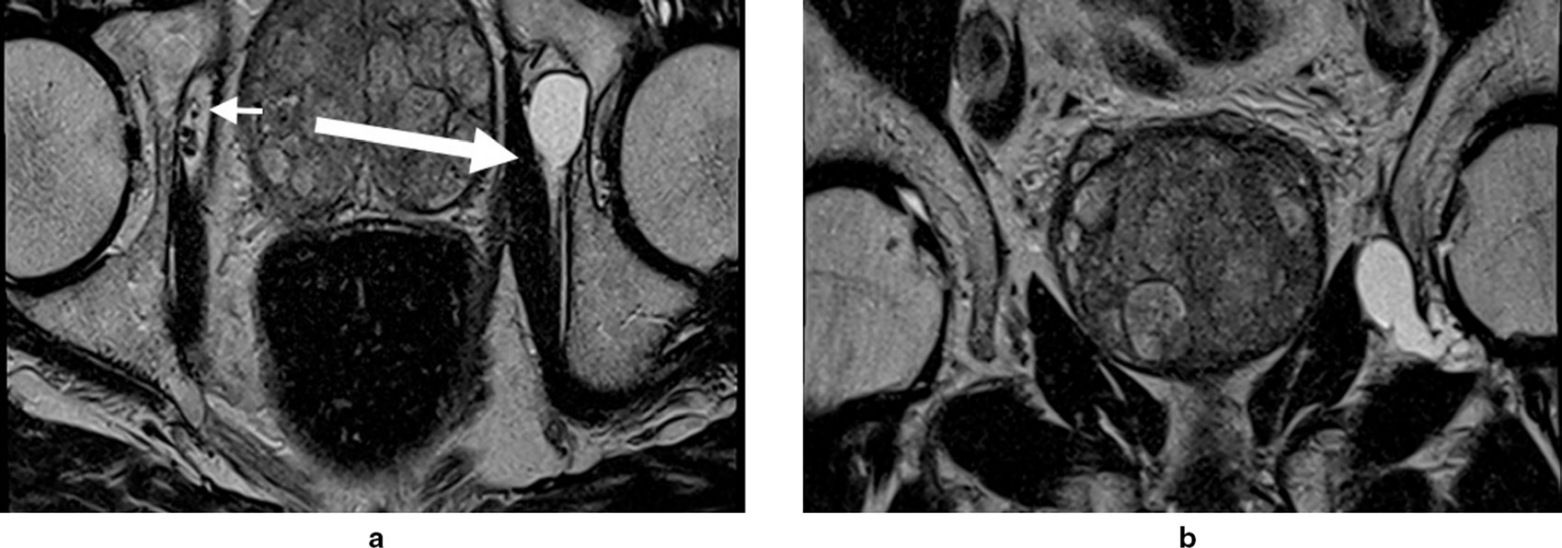
72-year-old male. Figure 4A (axial) and Figure 4B: (coronal) *T*_2_W images show a paralabral cyst arising medially from the left hip and extending alongside the obturator nerve (long arrow). The normal contralateral obturator nerve is identified for comparison (short arrow). *T*_2_W, *T*_2_ weighted.

**Figure 5.**
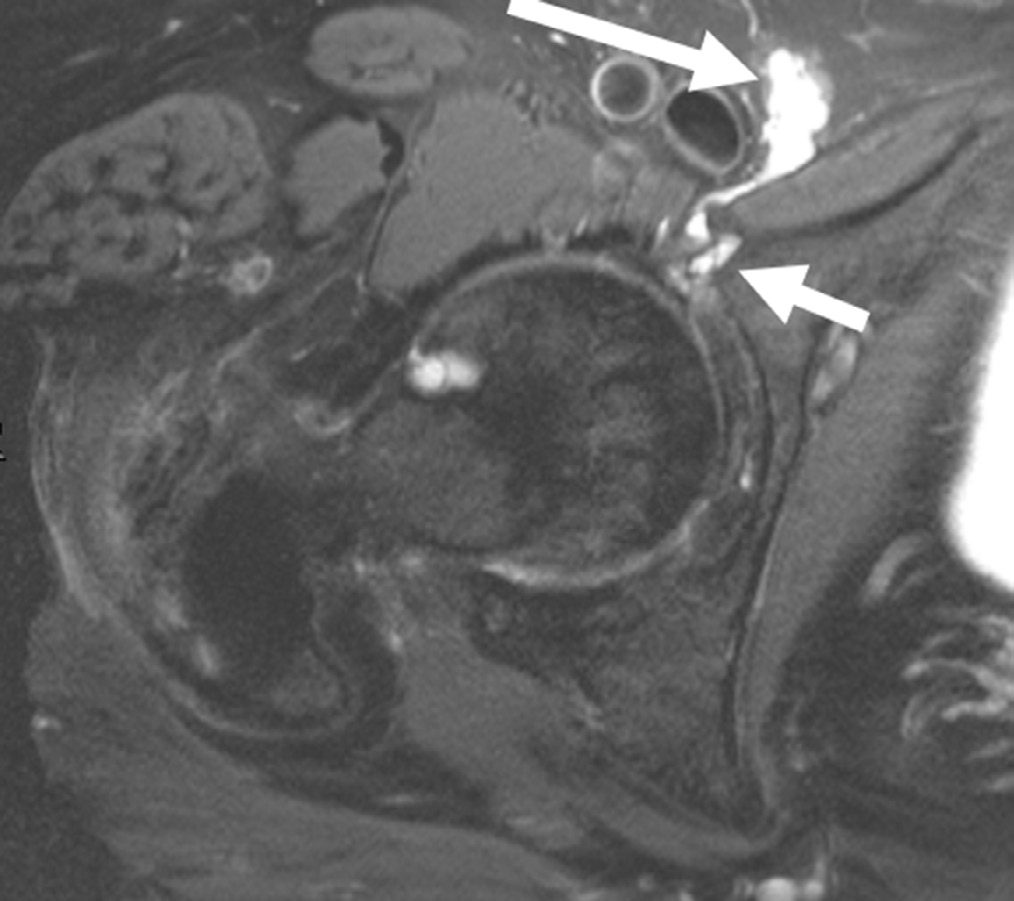
62-year-old male with right hip FAI and fibrocystic change at the femoral head-neck junction. Axial fsPDW image shows an anterior labral tear (small arrow) with a paralabral cyst propagating alongside right femoral nerve (long arrow). FAI, femeroacetabular impingement; fsPDW, fat saturated proton density-weighted.

**Figure 6.**
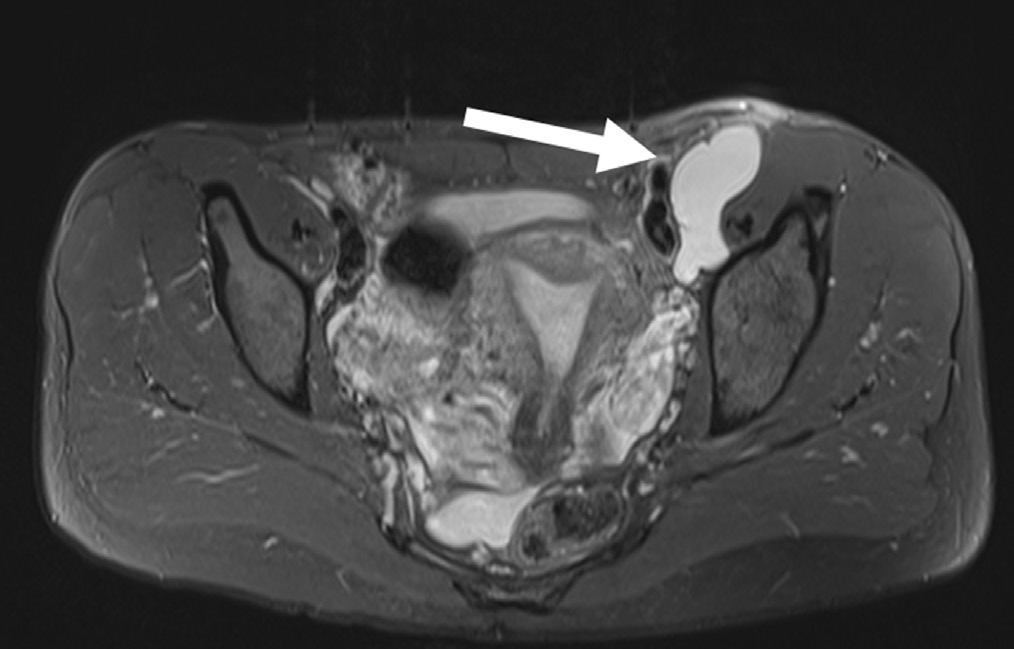
31-year-old female with left hip pain. Axial STIR image demonstrates a lobulated paralabral cyst arising anteriorly from the left hip joint and displacing the femoral neurovascular bundle. STIR, short tau inversion recovery

The cysts were multilocular in all cases and were homogenously T2 hyperintense in 13/14 (92.9%), with heterogenous T2 signal in one case (7.1%). The most common location with respect to the hip joint was posterior (7/14, 50.0%, see Figures 1–3) followed by medial (4/14, 28.6%, see Figure 4). The remainder of the cysts were anterior (2/14, 14.3%, see Figures 5–6)) and superior (1/14, 7.1%) with regard to the hip joint. Labral tears were identified in 11/14 cases (78.6%). The sciatic nerve was most involved in 5/14 cases (35.7%) with the obturator (4/14, 28.6%), femoral nerve (2/14, 14.3%), medial femoral cutaneous nerve (1/14, 7.1%), superior gluteal nerve (1/14, 7.1%) and inferior gluteal nerve (1/14, 7.1%) also affected. None of the patients had evidence of regional muscle denervation edema and/or atrophy.

### Interventions and outcomes

No intervention was undertaken in 9/14 cases (64.3%). 4/14 (28.6%) patients underwent CT-guided fenestration and aspiration with corticosteroid injection, with one of those patients subsequently proceeding to arthroscopic decompression. 1/14 patients (7.1%) underwent ultrasound-guided aspiration and corticosteroid injection. 4/5 patients who underwent intervention reported reduced pain at short-term (3 months) follow-up following the procedure while the fifth patient reported no change in symptoms. Long-term outcomes were not available.

Characteristics of the cysts, patient demographics and details of interventions are fully depicted in Table 1.

**Table 1.** Describes patient demographics, imaging characteristics of the paralabral cysts and intervention undergone where applicable.

Case	Age	Gender	Side	Predominant location	Labral tear present or absent	Affected nerve	T2 signal characteristics	Maximum dimension (cm)	hip dysfunction	Perineal pain	Groin pain	Hip pain	Intervention?	Effect of treatment?
1	62	F	Right	Anterior	Anterior	Medial femoral cutaneous nerve	Homogeneous	3.4	N	N	N	Y	No intervention	N/A
2	63	F	Left	Posterior	Absent	Superior gluteal nerve	Homogeneous	4.1	N	N	N	Y	No intervention	N/A
3	72	M	Left	Medial	Anterior	Branch of obturator nerve	Homogeneous	3.3	N	N	N	N	No intervention	N/A
4	46	F	Left	Posterior	Absent	Sciatic nerve	Homogeneous	3.7	N	N	N	Y	CT guided fenestration and partial aspiration	Reduced pain
5	79	M	Left	Medial	Anterior	Obturator nerve	Homogeneous	5.3	N	N	N	N	No intervention	N/A
6	73	M	Right	Superior	Anterior	Femoral	Homogeneous	3.9	N	N	N	N	No intervention	N/A
7	40	M	Right	Posterior	Posterior	Sciatic nerve	Homogeneous	3	N	N	N	Y	No intervention	N/A
8	56	M	Right	Posterior	Absent	Sciatic nerve	Homogeneous	4.8	N	Y	Y	Y	CT guided fenestration and aspiration then arthroscopic decompression later	Reduced pain
9	73	M	Left	Posterior	Posterior	Inferior gluteal nerve	Homogeneous	5.1	N	Y	N	Y	CT guided fenestration and aspiration	Reduced pain
10	56	M	Left	Posterior	Posterior	Sciatic nerve	Homogeneous	3.1	N	N	N	Y	No intervention	N/A
11	31	F	Right	Anterior	Anterior	Femoral	Homogenous	6.1	N	N	Y	Y	US guided aspiration	Reduced pain
12	30	F	Right	Posterior	Posterior	Sciatic nerve	Homogeneous	2	N	N	N	Y	No intervention	N/A
13	45	M	Left	Medial	Medial	Obturator nerve	Homogeneous	2.5	N	N	Y	N	No intervention	N/A
14	71	M	Left	Medial	Multifocal degenerative tears	Obturator nerve	Heterogeneous	7.3	Y	N	N	Y	Ct guided aspiration	None

## Discussion

The acetabular labrum is a thin fibrocartilaginous ring that deepens the acetabulum increasing the surface area, protects the hyaline cartilage, and contributes to hip joint stability and range of motion.^6^ Acetabular paralabral cysts are synovial cysts—sacs containing synovial fluid, lined by the synovial membrane. While the exact pathogenesis of paralabral cysts is somewhat controversial, they are usually seen in association with labral tears and often in the setting of degenerative disease.^2^ They have also been described in series of patients with hip dysplasia and femeroacetabular impingement (FAI).^7^ It is estimated that up to 50–70% of patients with labral tears have a paralabral cyst at MRI.^8^ This association supports the hypothesis that labral abnormalities are responsible, with synovial fluid escaping through the labral tear into a herniation of the synovial membrane.^5^ In our study, 78.6% patients had labral tears. MRI is thought to be relatively insensitive in the diagnosis of labral tears, yielding a positive result in only 36% of cases in certain series.^8^ MR arthrography is the gold-standard allowing excellent evaluation of the labrum and being positive in 91% of cases with a confirmed labral tear at surgery.^9^

Acetabular paralabral cysts are often clinically silent, having been described in up to 26.2% of asymptomatic hips.^10^ This was also seen in our series of larger cysts demonstrating perineural propagation, where 21.4% patients had no attributable symptoms. Paralabral cysts can produce pain (which may be severe) and variable mechanical symptoms have been described. In many instances, it is difficult to entirely elucidate symptoms due to the cyst and those contributed to by the underlying labral abnormality.

Compression of periarticular structures, typically nerves, by paralabral cysts has been described but only in a handful of case reports.^2–5,11^ Symptomatic compression of the sciatic, femoral and obturator nerves has also been described as well as the femoral vein.^3^ Several patients in our series presented with symptoms which may be attributable to nerve compression.

**Figure 7.**
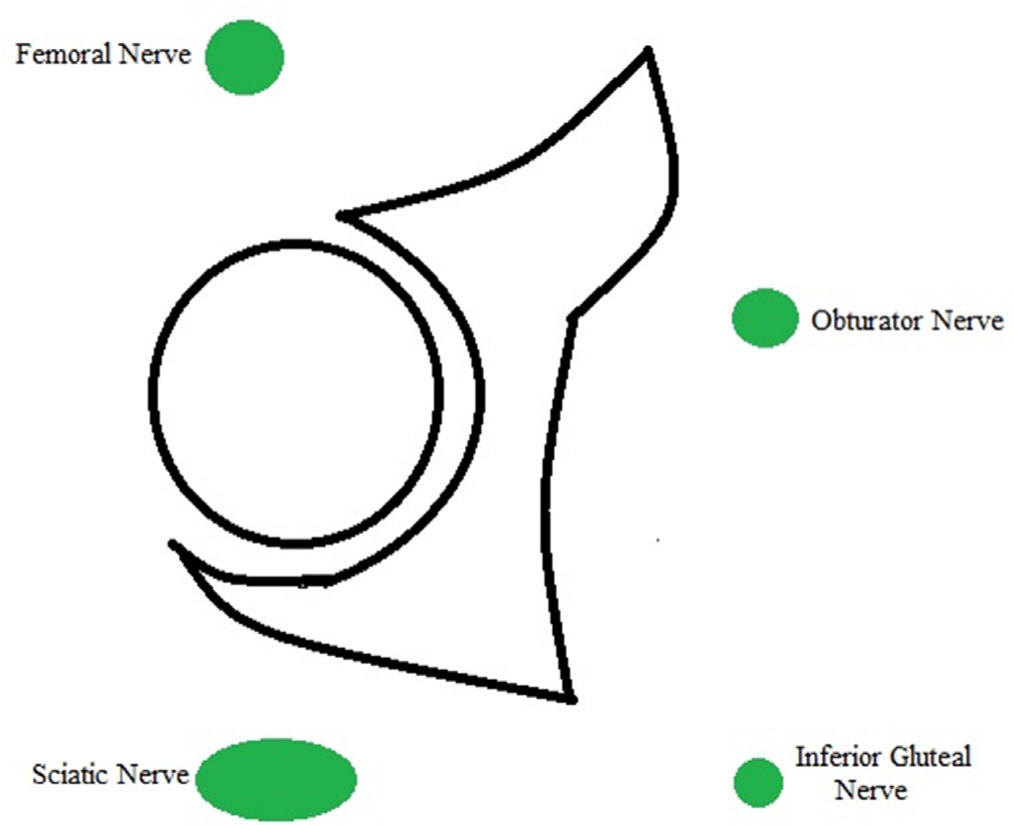
Schematic diagram demonstrating the relationship between the (left) hip joint and major nerves in the vicinity.

MRI is the definitive modality in the evaluation of periacetabular cystic lesions due to its unparalleled soft tissue contrast, which allows accurate diagnosis in most cases and assists with differentiation from similar appearing but more sinister entities, such as para-articular synovial sarcoma. 1/14 cases in our series (7.1%) demonstrated heterogenous T2 characteristics which can mimic aggressive entities. MRI also affords examination of the relationship between the paralabral cyst and the major nerves in the vicinity of the hip joint. As with many areas in musculoskeletal imaging, precise knowledge of anatomy is crucial to the interpretation of such relationships. Figure 7 provides a schematic overview of the relationship between the hip joint and the major nerves in this region. The obturator nerve arises from the anterior divisions of L2-4 in the lumbar plexus. It descends medial to the psoas major muscle and enters the obturator canal. Here, it may be vulnerable to compression by paralabral cysts arising medially from the acetabulum, as is depicted in Figure 4. The sciatic nerve forms from the anterior divisions of L4-S3 and the posterior divisions of L4-S2. In its proximal portion, it passes just posterior to the ischium and here may be subject to compression by paralabral cysts arising posteriorly from the hip. Figures 2 and 5 depict paralabral cysts propagating along the sciatic nerve. The femoral nerve arises from the posterior divisions of the L2-4 roots of the lumbar plexus. In the femoral triangle, it runs just outside and lateral to the femoral sheath and here may be compressed by paralabral cysts arising anteriorly from the hip. Figure 6 depicts a paralabral cyst propagating along the left femoral nerve and displacing the femoral vascular structures. The medial femoral cutaneous nerve is a branch of the superficial division of the femoral nerve. Figure 1 depicts a paralabral cyst propagating along the medial femoral cutaneous nerve. The superior and inferior gluteal nerves arise from the posterior divisions of the sacral plexus and exit the pelvis by passing through the greater sciatic foramen, above and below the piriformis muscle, respectively. Figure 3 depicts a paralabral cyst propagating along the inferior gluteal nerve.

While there is a paucity of evidence regarding treatment options for symptomatic paralabral cysts at the hip joint, ultrasound-guided aspiration and surgical excision have historically formed the mainstay of treatment.^12^ Aspiration—with or without corticosteroid injection—allows symptomatic relief however recurrence is relatively common, occurring in up to 50% of cases.^13^ Surgery allows both excision of the offending cyst and the opportunity for repair of the underlying labral abnormality.^14^ Arthroscopic excision of paralabral cysts is safe and effective and is associated with excellent clinical outcomes.^15^

Our study has several limitations. It is difficult to be certain of the exact contribution of the perineural propagation of a paralabral cyst to an individual patient’s symptoms, as evidenced by the fact that some patients were asymptomatic. The improvement of symptoms following intervention in several instances suggests that there is a potential for symptomatology, however. Furthermore we were unable to gather data on the long-term outcomes following intervention, and there is a possibility that even those patients who experienced an improvement initially may have subsequently had recurrent symptoms.

In summary, paralabral cysts may uncommonly demonstrate perineural propagation along major nerves in the vicinity of the hip joint. Such cases have a varied clinical presentation. They may extend into the periacetabular tissues and can demonstrate perineural extension. Most patients who underwent image-guided or surgical interventions reported an improvement in symptoms.
